# Formation of functional super-helical assemblies by constrained single heptad repeat

**DOI:** 10.1038/ncomms9615

**Published:** 2015-10-15

**Authors:** Sudipta Mondal, Lihi Adler-Abramovich, Ayala Lampel, Yaron Bram, Sophia Lipstman, Ehud Gazit

**Affiliations:** 1 Department of Molecular Microbiology and Biotechnology, George S. Wise Faculty of Life Sciences, Tel Aviv University, Tel Aviv 69978, Israel; 2 Department of Oral Biology, The Goldschleger School of Dental Medicine, Tel Aviv University, Tel Aviv 69978, Israel; 3 School of Chemistry, Sackler Faculty of Exact Sciences, Tel Aviv University, Tel Aviv 69978, Israel; 4 Department of Materials Science and Engineering, Iby and Aladar Fleischman Faculty of Engineering, Tel Aviv University, Tel Aviv 69978, Israel

## Abstract

Inspired by the key role of super-helical motifs in molecular self-organization, several tandem heptad repeat peptides were used as building blocks to form well-ordered supramolecular nano-assemblies. However, the need for stable helical structures limits the length of the smallest described units to three heptad repeats. Here we describe the first-ever self-assembling single heptad repeat module, based on the ability of the non-coded α-aminoisobutyric acid to stabilize very short peptides in helical conformation. A conformationally constrained peptide comprised of aromatic, but not aliphatic, residues, at the first and fourth positions formed helical fibrillar assemblies. Single crystal X-ray analysis of the peptide demonstrates super-helical packing in which phenylalanine residues formed an ‘aromatic zipper' arrangement at the molecular interface. The modification of the minimal building block with positively charged residues results in tight DNA binding ascribed to the combined factors of helicity, hydrophobicity and charge. The design of these peptides defines a new direction for assembly of super-helical nanostructures by minimal molecular elements.

## Abstract (web summary)

Advances in bionanotechnology demand an increased portfolio of assemblies beyond those currently available. Here, the authors design a crystallographically characterized super-helical sequence composed of single heptad repeats which, through derivatisation, offers vast potential applications.

## Graphical abstract

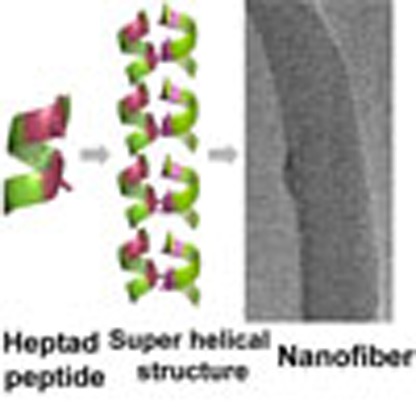

The formation of ordered nanostructures by small peptide building blocks is a key direction in bionanotechnology^1^,^2^,^3^,^4^. It was demonstrated that minimal peptide modules, as short as dipeptides, could formed ordered structures at the nanoscale^5^,^6^,^7^. However, the formation of assemblies by such short peptides is limited mainly to β-sheet organization of the peptide motifs in the formed nanostructures^8^,^9^,^10^,^11^. While these assemblies offers remarkable mechanical, optical, piezolelctric and semiconductive proprieties, they lack the precise orientation of amino-acid residues needed for directed intermolecular organization and specific interactions with target molecules such as DNA or other biopolymers^12^,^13^. Indeed, in many biological systems, the recognition of DNA is facilitated by ordered super-helical structural motifs^14^,^15^,^16^. This super-helical organization commonly comprised of two or more individual α-helices that interact with each other via hydrophobic facets present in protein surfaces and oligomerized to bundled structure. The canonical helical segment is composed of a recurring sequence of seven amino acids known as a heptad repeat and designated as ***abcdefg***, where positions ***a*** and ***d*** are often occupied by hydrophobic amino acids such as leucine, isoleucine and valine.

The central biological function of super-helical proteins, and the fact that this module is one of the few motifs where amino-acid sequence rules can predict higher order interactions among individual secondary structures, afforded the design of several super-helical assembly building blocks^17^,^18^. In nature, the length of such proteins ranges from just a few heptad repeats to very large segments as long as 1,000 amino acids and they usually exist as discrete structures. Several *de novo* design principals were adopted to promote propagation of super-helical motif to fibrous structures. In a pioneering study, Woolfson *et al.* developed the ‘sticky-ends' design by placing complementary charged amino acids at position ***e*** and ***g*** of heptad repeat of adjacent helical peptides. The 28 residue peptides, having four heptad repeats, formed staggered heterodimers with overhanging ends. The presence of complementary core and ion-pair interactions at the overhang positions facilitates longitudinal association of the dimers to afford helical assemblies. Finally, lateral assembly of that structure afforded nanofibres^19^,^20^. In another study, Hartgerink and colleagues designed the shortest three heptad long peptide sequences without ‘sticky-end' features and showed that the blunt-ended dimers also have the ability to undergo longitudinal and lateral assemblies to form nanofibres^21^. Several others peptides that have the propensity for helical super structure formation were designed by modifying the nature of hydrophobic amino acids in the core as well as the characteristics of the residual heptad positions. These designer building blocks self-assembled into distinct nanostructures having morphology ranging from fibres to net-like architectures to vesicles^22^,^23^,^24^,^25^,^26^,^27^. Here we describe for the first time the design and formation of a super-helical structure formed by the molecular self-assembly of a single heptad peptide sequence. The minimal modification of the original sequence by the incorporation of a positively charged amino acid could modulate the zipper-like structure into a functional DNA-binding module.

## Results

### Peptide design

**Figure 1:**
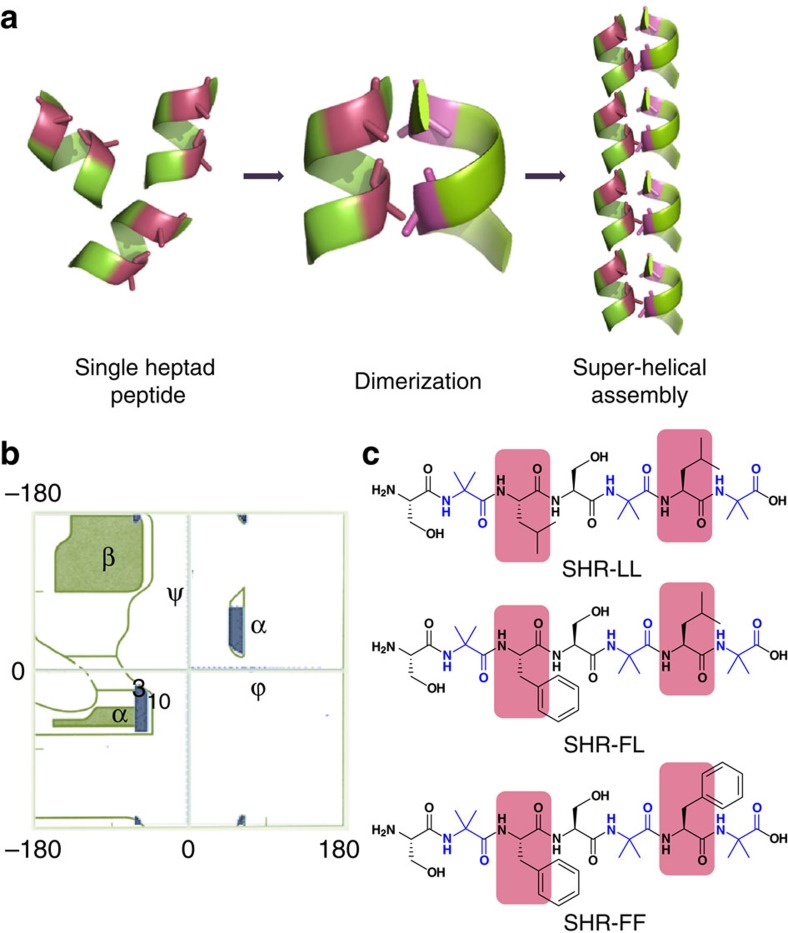
Proposed model and design of super-helical sequence composed of single heptad repeats. (**a**) Depiction of helical single heptad peptide module in which the positions ***a*** and ***d*** are displayed in pink colours. Assembly of these single heptad modules via dimerization may afford the formation of super-helical assembly. (**b**) Ramachandran plot of the sterically allowed dihedral angles of Aib residues, showing preference for helical conformation (blue colour region). (**c**) Chemical structure of the designed peptides in which Aib residues are illustrated in blue and ***a*** and ***d*** positions of heptad repeats are shaded with pink.

In an alternative design strategy, we envisioned that the presence of multiple heptad repeats may not be a prerequisite for the formation of helical supramolecular assemblies and the aggregation of a conformationally constrained single heptad sequence could serve as the paradigm for construction of super-helical arrangement (Fig. 1a). We designed some seven residue monomeric peptides with hydrophobic amino acids at positions ***a*** and ***d***. The design scheme was based on the assumed association of the peptides via hydrophobic interactions to form dimers, which could be further elongated in the axial direction, by forming hydrogen bonds between the N- and C-termini of two dimeric units positioned on top of each other (Fig. 1a).

However, the organization of a seven residue peptide sequence into a three-dimensional helical fold is thermodynamically unfavourable^28^,^29^.To circumvent this challenge and stabilize the helical conformation in short sequences, we resorted to the use of the non-coded amino acid α-aminoisobutyric acid (Aib), an extension of alanine with a di-methylated α-carbon atom^30^. The incorporation of this amino acid has been demonstrated to efficiently stabilize short helical peptides produced by various microorganisms. The presence of an additional α-methyl group limited the ranges of allowed *ϕ*, *ψ* torsions angles of Aib compared with alanine and imposed restrictions over the energetically accessible conformational space. Figure 1b shows the permissible Aib conformation map compiled by superposition of the Ramachandran plots for L-Ala and D-Ala residues^31^,^32^. As evident from this figure, the allowed *ϕ*, *ψ* values of Aib are constrained within the range of ±60° (±20°) and ±30° (±20°), corresponding to the left-handed and right-handed helical region of the Ramachandran plot, respectively. This helix-inducing characteristic of Aib was exploited in our design strategy by incorporating several Aib residues at strategic locations in the monomeric heptad sequence (Fig. 1c).

The nature of the interfacial interactions that dictate the dimerization of the heptad repeat plays a pivotal role in stabilization of helical assemblies^33^,^34^.The favourable interactions in helical assemblies were integrated into our design by including two leucine residues in peptide SHR-LL (H_2_N-Ser-Aib-Leu-Ser-Aib-Leu-Aib-OH; Fig. 1c). To further strengthen the interactions at the domain core, we sequentially substituted leucine with phenylalanine, which is significantly more rigid and can participate in aromatic stacking^5^. Such structural features exist at the N-terminal homology domain of APS protein, where stacking of 10 phenylalanine residues within the hydrophobic core of the dimerization domain creates a new structural motif termed the ‘phenylalanine zipper'^35^. Following these structural insights, two additional peptides SHR-FL (H_2_N-Ser-Aib-Phe-Ser-Aib-Leu-Aib-OH) and SHR-FF (H_2_N-Ser-Aib-Phe-Ser-Aib-Phe-Aib-OH) were designed (Fig. 1c). Assemblies of these peptides were explored using different spectroscopic methods, electron microscopy and X-ray crystallography.

### Secondary structures and self-assembly of the peptides

**Figure 2:**
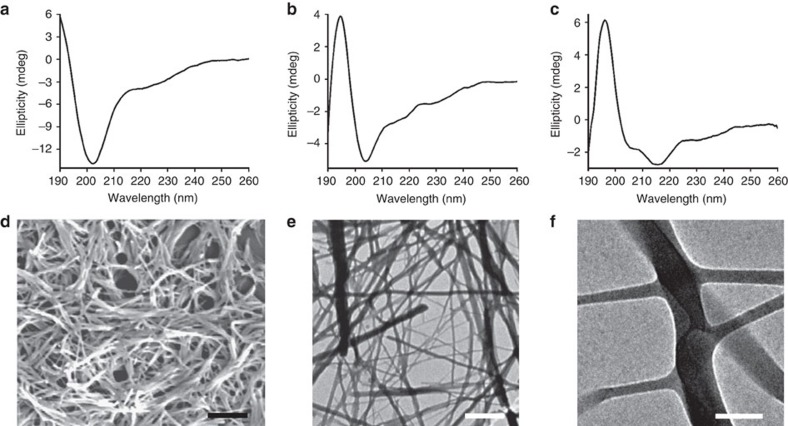
Structural analysis and self-assembly of Aib containing peptides. CD spectra of the designed peptides: (**a**) SHR-LL, (**b**) SHR-FL and (**c**) SHR-FF. (**d**) SEM micrograph of the peptides SHR-FF. Scale bar, 2 μm. (**e**) TEM micrograph of the peptides SHR-FF. Scale bar, 1 μm. (**f**) High resolution TEM images showing the presence of branching along the length of the fibres. Scale bar, 100 nm.

We studied the solution conformation of the peptides by circular dichroism (CD) spectroscopy in phosphate buffer at pH=7.4. SHR-LL exhibited a negative maximum at around 201 nm, corresponding to the unstructured or random coil conformation (Fig. 2a). Peptide SHR-FL, where a single leucine residue was replaced with phenylalanine, showed a red shifting of the negative ellipticity to 205 nm with simultaneous appearance of a positive peak near 195 nm (Fig. 2b) and may suggest the presence of random coil or 3_10_ helical conformations. Surprisingly, only the SHR-FF peptide containing phenylalanine at both ***a*** and ***d*** positions showed a classical double-negative-maxima CD signal, characteristics of a helical conformation. The peptide exhibited two negative maxima at around 205 and 218 nm and a positive maximum at 195 nm (Fig. 2c). These signals are slightly blue shifted compared with the ideal α-helical peptides (208 and 222 nm), but showed similarity with reported short helical sequences. Fairlie and colleagues showed that the CD signals of single turn α-helices usually appears at 207 and 215 nm and this is consistence with the long wavelength n–π* transition commonly observed for α-helices and β-sheets in the 215–230 nm wavelength range^36^. In another study, Baldwin and colleagues have showed that the negative maxima at 222 nm shifts to 219, 217 and 215 nm as the sequence becomes shorter from 11 to 8 to 4 helical peptide units^37^. These studies support that the two negative maxima at 205 and 218 nm along with a positive maximum at 195 nm for SHR-FF arises due to the helical conformation of the peptide in solution. The stability of the SHR-FF backbone conformation was assessed by temperature dependent denaturation experiments. As shown in Supplementary Fig. 1, the peptide underwent thermal unfolding on increasing the temperature, but maintained residual helicity even at 90 °C. In addition to that, the peptides regained the original conformation on cooling, suggesting the exceptional conformational stability of SHR-FF.

The differences in secondary structure among the designed peptides inspired us to study the self-assembly properties of the three peptides in solution. We used electron microscopy to obtain further information regarding the morphology of the self-assembled peptides. The SHR-LL and SHR-FL peptides, which did not undergo defined folding patterns, exhibited amorphous aggregates or layered structures without well-organized morphology (Supplementary Fig. 2a,b). Analysis of the SHR-FF peptide, which adopted a helical conformation, revealed the formation of fibre structures with long persistent length as shown in Fig. 2d. The diameters of the fibres were not uniform but maintained a narrow distribution range of 40–80 nm, and part of the assemblies underwent further aggregation to afford bundles of fibres (Fig. 2e). In addition, the appearance of branching at different segments of the fibres was also observed (Fig. 2f). The characteristic ellipticity and self-assembly of the SHR-FF peptide demonstrated that the presence of phenylalanine at positions ***a*** and ***d*** was a prerequisite for helical folding as well as for supramolecular organization of this designed peptide. We assumed that the planar rigidity and bulky structure and the tendency for geometrically restricted interactions between the phenylalanine residues may contribute to the ability of this moiety to facilitate the ordered molecular self-assembly as compared to the aliphatic leucine^6^,^8^,^13^.

### Crystal structure of SHR-FF

**Figure 3:**
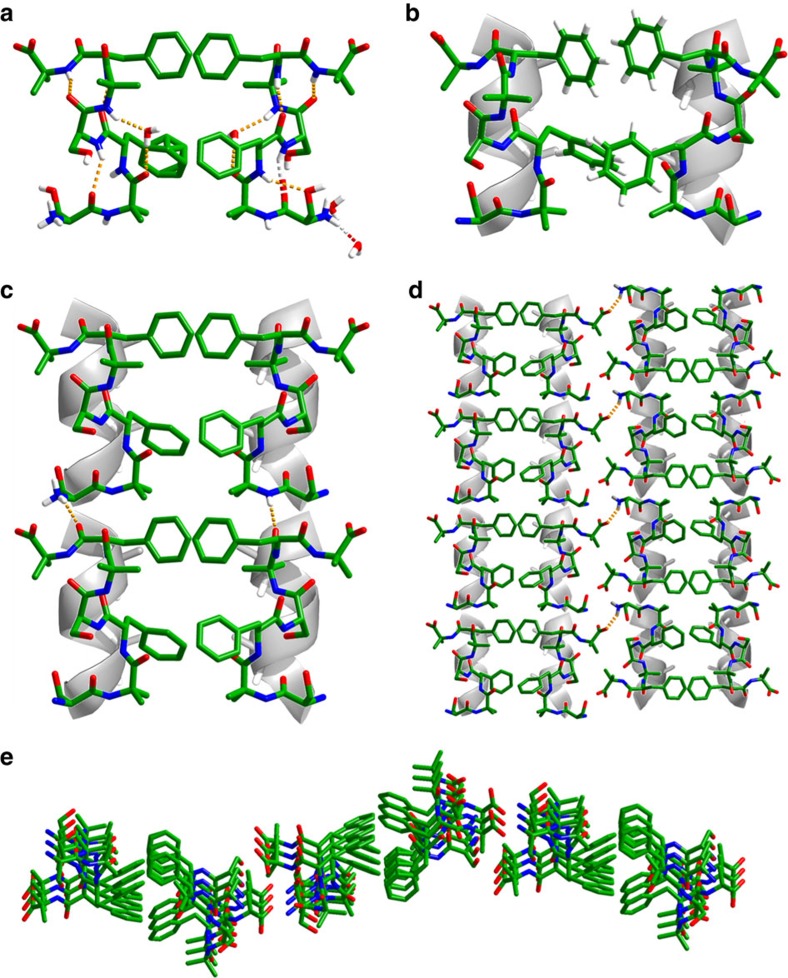
Single crystal X-ray analysis of SHR-FF. (**a**) Two different conformations of the molecules present in the asymmetric units. (**b**) Aromatic–aromatic interactions stabilized the dimeric interface of the conformers. (**c**) Molecular packing of the peptide creates zipper-like structures in which hydrogen bonds (shown by yellow dotted lines) connect the individual dimers. (**d**) Antiparallel packing of adjacent zipper modules. (**e**) View of three adjacent zipper-like structures from the C-terminus of the SHR-FF super-helical organization.

To gain further structural insights and to elucidate the favourable interactions that led to supramolecular organization, we grew crystals of the SHR-FF peptide in phosphate buffer at pH 7.4, the identical solution conditions used for the CD and the self-assembly studies. The asymmetric unit of SHR-FF crystal was comprised of two peptide molecules and several ordered or disordered water molecules. The two molecules in the SHR-FF asymmetric unit shared common structural features and revealed i and i+3 hydrogen bonding patterns reminiscent of 3_10_ helical structures (Fig. 3a). However, the hydrogen bonding was not continuous and a single water molecule invaded the backbone. The water molecule formed hydrogen bonds with both NH and CO groups located at the i and i+3 positions and had the potential to undergo backbone hydrogen bonding. The backbone torsion angles of all the amino-acid residues with the exception of phenylalanine corresponded to the right-handed 3_10_ helical conformation of the Ramachandran map, whereas the *ϕ* and *ψ* of the four phenylalanine residues were −99.27, −110.64, −97.27, −105.96 and 22.86, −4.32, 28.40, 0.1, respectively (Supplementary Fig. 3). These distinct dihedral angles granted the SHR-FF peptide a unique conformation in which a type III turn, corresponding to the single turn of a classical 3_10_ helix, was flanked by two type I turns^38^. The average *ϕ* and *ψ* of the peptide were −75° and −15°, respectively, and these values were in good agreement with the canonical 3_10_ helix originally proposed by Pauling (canonical *ϕ* and *ψ* are −74° and −4°, respectively)^39^. Two molecules of SHR-FF interacted with each other in a parallel orientation to form a dimeric unit as shown in Fig. 3b (to aid the visualization, the peptides were superimposed over an ideal helical model). The interface of the dimer was stabilized by the intermolecular interactions between the phenylalanine side chains as envisioned in the original design and revealed two different types of arrangements corresponding closely to the T-shaped and parallel displaced structures commonly observed in helical proteins and peptide crystals. These dimers propagated along the *c* axis of the crystal to create a continuous array of amphiphatic arrangements of phenylalanine side chains connected mainly by head-to-tail backbone hydrogen bonds, involving both the terminal amine group and internal amide bonds (Fig. 3c). These columnar associations can be considered as the supramolecular packing of the 3_10_ helices anchored by two different turn segments, somewhat resembling the proposed self-assembly model of β-sheet forming tripeptide ^D^LFF (ref. 40). Although the dimer shows parallel packing of SHR-FF peptides, two adjacent layers of SHR-FF dimers adopt an antiparallel orientation relative to the amino-to-carboxyl dipole of each column and form hydrogen bonds between the free NH and CO groups present at the termini, as shown in Fig. 3d. This antiparallel arrangement of the molecules is favourable considering the fact that the dipole of one column can eliminate the opposing dipole of the adjacent column. Figure 3e represents a different perspective of the arrangement of the peptides in the crystal and reveals that the parallel–antiparallel packing of the peptide columns was not linear, but rather displayed a curved pattern. The packing of the curve layers on top of each other involving hydrogen bonding and *π*-stacking may lead to the formation of fibre structures (Supplementary Fig. 4).

### Characterization of fibres and possible assembly pathway

To understand the conformational preference of the SHR-FF in fibrillar morphology, Fourier transform infrared (FTIR) spectroscopy was employed. The spectra show three distinct vibrational frequencies at 1,643, 1,665 and 1,673 cm^−1^ in the amide I region and can be correlated with the presence of type III and type I turns, a topography matching solid state structure (Supplementary Fig. 5c)^41^. This experiment suggests that the conformation of the SHR-FF peptides was similar in both the crystal structure and in fibres. FTIR of SHR-LL and SHR-FL were recorded under similar conditions and displayed broad signals over the entire range of the amide I region, indicating the inherent flexibility in the backbone conformation (Supplementary Fig. 5a,b). These observations can partly explain the apparent inabilities of these peptides to form self-assembled structures. We proposed a tentative model based on these experimental evidences to account for the formation of fibrillar structures by SHR-FF and its salient features (Supplementary Fig. 6). The dimeric species stabilized by interacting hydrophobic interfaces would laminate along the *c* axis to form a zipper-like monolayer and would propagate along the *a* and *b* axes to form multilayer structures involving mostly H-bonding and stacking interactions. It can be speculated that the long axis of the fibres coincide with the *c* axis and the widths of the fibres would correspond to the parallel or antiparallel packing of the zipper modules, as interactions with solvents can mitigate the stability associated with antiparallel packing observed in crystals. The presence of budding or kinking on fibre surfaces (Supplementary Fig. 6, white circle) can induce a secondary nucleation process along the a or b directions leading to branching as observed in transmission electron microscopy (TEM). This proposed model suggested that the presence of uncapped N- and C-terminus is significant for the propagation of a dimeric motif into fibrillar assemblies. To verify these assumption, we examined the secondary structural conformation and self-assembly of protected SHR-FF (Ac-SHR-FF-NH_2_). The protected peptide showed similar backbone conformation as evident from CD spectroscopy; however, it did not reveal any well-defined self-assembly morphology (Supplementary Fig. 7). This observation validated our strategy that a well-designed single heptad repeat in conjugation with free end-termini are necessary requirements for the realization of a supramolecular helical assembly and it also explains the present inaccessibility of self-assemble structures of Aib peptides, which have been studied mostly in protected sequences.

### Modifications of heptad peptide into DNA-binding sequence

**Figure 4:**
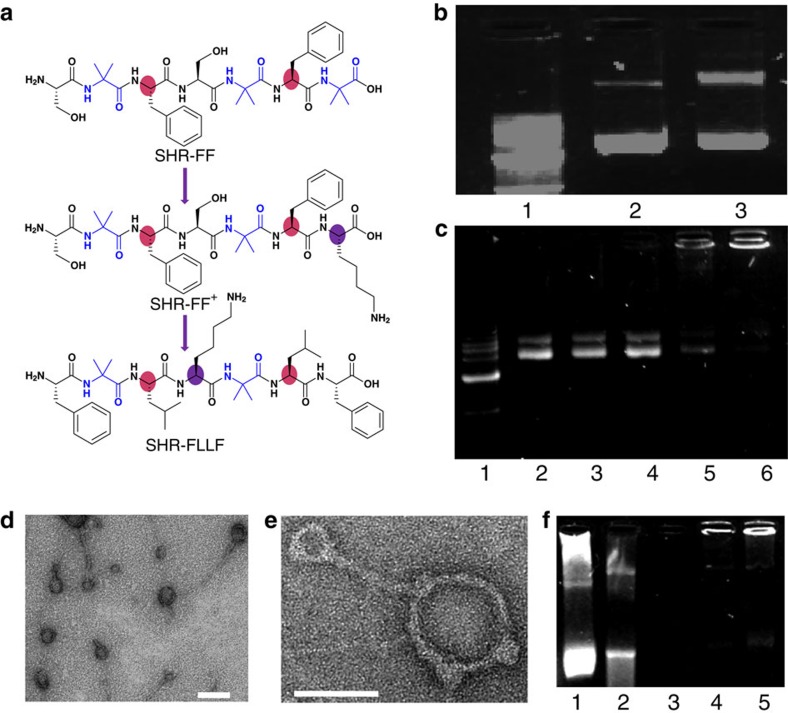
Structural modification and DNA condensation by modified single heptad peptide. (**a**) Modification of the SHR-FF sequence to afford the positively charged single heptad repeat module containing single lysine residue. Colour regions display the significant variation in amino-acid residues. (**b**) Agarose-gel electrophoretic assay of DNA condensation by SHR-FF^+^. Lane 1: 1 kb marker; lane 2: free DNA; lane 3: N/P 100. (**c**) Agarose-gel electrophoretic assay of DNA condensation by SHR-FLLF. Lane 1: 1 kb marker; lane 2: free DNA; lane 3: N/P 1; lane 4: N/P 20; lane 5: N/P 50: lane 6; N/P 100. (**d**) TEM images of complexes of plasmid DNA with SHR-FLLF (N/P 100). Scale bar, 100 nm. (**e**) Magnified TEM image showed the presence of rod-like and toroidal structures of a peptide-DNA complex. Scale bar, 50 nm. (**f**) DNase I digestion assay. Lane 1: free DNA without DNase I; lanes 2–3: free DNA treated with DNase I for 5 and 30 min, respectively; lanes 4–5: DNA-peptide (N/P 100) complex treated with DNase I for 5 and 30 min, respectively (N/P is the ratios between positive charges of peptide to negative charges of phosphate groups in DNA).

Leucine-zipper proteins are comprised of two different segments: the dimerization domain and the DNA-binding domain^42^. The formation of a preferential dimeric super-helical structure of the SHR-FF peptide motivated us to explore the possibility of designing a single heptad repeat-based DNA-binding module. In addition to the helical secondary structure, DNA-binding peptide sequences are usually composed of positively charged amino acids that facilitate electrostatic interactions with the negatively charge DNA backbone. The SHR-FF peptide was modified by the inclusion of positively charged lysine at C-terminus to afford SHR-FF^+^. Lysine was preferably placed at that location as positively charge capped C-terminus induce higher helicity (Fig. 4a)^43^. The DNA condensing properties of this peptide was evaluated by complexing SHR-FF^+^ with pEGFP-C2 plasmid DNA. The electrophoretic mobility assay showed considerable interactions between the positively charged sequence and the plasmid DNA, but failed to exhibit optimal condensing efficiency even at high concentrations and the presence of residual DNA band can be observed (Fig. 4b). Literature reports suggested that the hydrophobic forces and *π*–*π* interactions can complement the essential electrostatic interactions associated with DNA-peptide condensation^44^,^45^. This observation afforded the designing of SHR-FLLF in which the phenylalanine residues was placed at termini and it was assumed that the conformational freedom of these terminal aromatic residues may impose *π*-stacking between the nucleobases and the minimalistic peptide modules (Fig. 4a). The helix favoring leucine was embedded at ***a*** and ***d*** positions of the heptad repeat and the positively charged lysine was included in place of serine (Fig. 4a). It is worth noting that a continuous stretch of three or more lysines are preferred for DNA binding^46^,^47^. Yet, the modified SHR-FLLF sequence consisted of only a single lysine residue, but we considered that the combined effects of helicity, hydrophobicity, *π*-stacking, and electrostatic interactions may facilitate SHR-FLLF to act as an efficient DNA condensing agent.

The secondary structure of the designed SHR-FLLF peptide was probed by FTIR spectroscopy and the presence of peaks at 1,653 cm^−1^, 1,663 cm^−1^ confirmed that the peptide adopted a helical backbone conformation that resembled the original SHR-FF sequence (Supplementary Fig. 8). The ability of SHR-FLLF to afford DNA condensation was validated by the complexation of a pEGFP-C2 plasmid DNA with peptide at different stoichiometric ratios. The agarose-gel electrophoretic mobility assay of the complex revealed that complete retardation of the DNA migration could be achieved by attainment of an N/P ratio of 50 (N/P is the ratios between positive charges of peptide to negative charges of phosphate group; Fig. 4c). This observation substantiates our assumption that subtle interplay of helicity, non-covalent and charge interactions could afford efficient DNA condensation without the presence of several lysine residues in a single sequence. Further analysis of the morphology of the complex was performed by TEM. The distinctive formation of toroid and rod-like structure with diameters in the range of 50–100 nm indicated peptide induced condensation of plasmids (Fig. 4d). The appearance of such topology commonly occurs in the condensation phenomenon by polyamine species^44^,^48^. The co-existence of toroid and rod-like condensates could be observed for peptide-DNA complex with ratios of N/P 50 and N/P 100 with the predominant toroid shape in the latter. These characteristic features were not observed at lower N/P ratios (Supplementary Fig. 9). The increased abundance of toroid shapes at higher peptide ratios and the presence of nodular segments in toroidal structures, as shown in Fig. 4e, portended that the condensation pathway may be comprised of initial electrostatic interactions between plasmids and lysine residues followed by assembly of SHR-FLLF aided primarily by non-covalent interactions.

The efficient DNA condensation by SHR-FLLF was predicted to inhibit the digestion of DNA by DNase I enzymes. To assess the possible protection, condensed and bare DNA were incubated with DNase I and the effectiveness of enzyme-induced degradation was analysed by agarose-gel electrophoresis. The DNase assay was optimized to visualize the bare DNA for a few minutes after the incubation. As shown in Fig. 4f and Supplementary Fig. 10, the condensed DNA could be detected after a prolong incubation period whereas degradation of bare DNA was completed within a few minutes. The efficient condensation and superior protection of the DNA by SHR-FLLF peptide comprised of hydrophobic and aromatic blocks with a single lysine residue may help to delineate the role of positive charges and other non-covalent interactions towards DNA condensation and eventually may lead to the design of efficient peptide-based transfection agents having less positive charge density, an essential requirement for transfecting agents with lower cytotoxicity^49^.

## Discussion

The use of peptide building blocks represents a central direction in bionanotechnology. However, before the current work, the molecular self-assembly of short (<8 residues) peptides into ordered nanostructures was dominated by β-sheet secondary structures. The modular approach described here illustrates an efficient design strategy inspired primarily by natural super-helical sequences to accomplish a minimal peptide module comprised of only seven amino acids to afford a supramolecular helical assembly. The aromatic amino acids, but not the aliphatic residues, positioned at the ***a*** and ***d*** sites of the heptad peptide initiate dimerization and secondary *π*-stacking and H-bonding interactions laminate the dimeric peptides into self-assembled fibres. The presence of uncapped end-termini is a needed requirement for propagation of the dimeric units into a mature fibril, as the corresponding capped sequence failed to initialize the assembly process. This study corroborates our molecular design principles that the combination of natural α-amino acids with defined predispositions and minimal numbers of helix-inducing Aib were sufficient to afford supramolecular helical ensembles that are amenable to greater functional variations. Subtle modifications of the fibre forming heptad sequence by inclusion of positively charged amino acids converted the original heptad peptide into a DNA-binding module. To the best of our knowledge, the described single heptad DNA-binding module is the smallest peptide sequence with the potential for DNA condensation reported in literature. The single heptad repeat peptide module described here may help in deciphering the role of hydrophobic and *π*-stacking interactions in DNA condensation and, at the same time, can provide crucial information about the DNA condensation efficiency of two different positively charged amino acids, lysine and arginine, by appropriate sequence modifications.

The established conformationally constrained single heptad repeat presented here as a minimal assembly module should pave the way for the increased use of helical nano-fibres formed by the assembly of simple building blocks that are modular and amenable for structure modifications. The high resolution crystal structure of the helical assemblies will allow the accurate molecular design of new analogues of this motif. Substitution at different positions of the heptad sequence while maintaining the relative arrangements of hydrophobic amino acids and introduction of added functionality through side chain modifications can give rise to a vast repertoire of helical assemblies with possible applications in bionanotechnology and biomaterials.

## Methods

### General methods

All the peptides were purchased from Peptron, Inc. (South Korea). Fresh stock solutions were prepared by dissolving the peptides in dimethylsulphoxide (DMSO) and diluting the solution with phosphate buffer (pH=7.4) to a final concentration of 5 mg ml^−1^.

### Transmission electron microscopy

The peptides were dissolved in DMSO in 100 mg ml^−1^ concentration and diluted with phosphate buffer to maintain final concentration of 5 mg ml^−1^. The sample was aged for 48 h with frequent shaking. A 5 μl aliquot of this solution was placed on a 400 mesh copper grids. After 1 min, excess fluids were removed. For negative staining, the grid was stained with 2% uranyl acetate in water and after 2 min excess fluids were removed from the grid. Samples were viewed using a JEOL 1200EX electron microscope operating at 80 kV.

### Scanning electron microscopy

The peptides were dissolved in DMSO in 100 mg ml^−1^ concentration and diluted with phosphate buffer to maintain a final concentration of 5 mg ml^−1^. A 10 μl aliquot of the peptide solution was dried at room temperature on a microscope glass cover slip and coated with gold. Scanning electron microscopy (SEM) images were taken using a JSM JEOL 6300 SEM operating at 20 kV.

### Analyses of secondary protein structure using CD

The peptides (5 mg ml^−1^) were dissolved in phosphate buffer (pH=7.4) and incubated for 1 h before the measurement of secondary structure using CD. The solutions were diluted to a final concentration 0.23 mM with original buffer and the samples were placed in a 1 or 0.1 mm cuvette. CD spectra in the range of 190–260 nm were recorded on a Chirascan spectrometer. Background was subtracted from the CD spectra. Temperature experiments were conducted between 20 and 90 °C. The samples were equilibrated for 10 min at the desired temperature before carrying out the CD measurement. CD spectroscopy of Ac-SHR-FF-NH_2_ was recorded by preparing the stock solution in hexafluoro-2-propanol and diluting with phosphate buffer to a final concentration of 5 mg ml^−1^.

### FTIR spectroscopy

FTIR spectra were collected using a Nicolet Nexus 470 FTIR spectrometer with a deuteratedtriglycine sulfate detector. The peptides were dissolved in DMSO to 100 mg ml^−1^ concentration and diluted with phosphate buffer to maintain final concentration of 5 mg ml^−1^. The sample was aged for 48 h with frequent shaking. A 30 μl aliquot of the peptide solution was deposited on a polyethylene IR card and dried under vacuum. The samples were saturated with 30 μl of D_2_O for two times and vacuum dried. Measurements were taken using a 4 cm^−1^ resolution and by averaging 64 scans. The absorbance maxima values were determined using an OMNIC analysis program (Nicolet). The background was subtracted using a control spectrum. FTIR spectrum of SHR-FLLF was recorded in phosphate buffer without the addition of D_2_O.

### Crystal structure determinations

The X-ray measurements (Nonius-Kappa CCD and Bruker-Apex Duo diffractometers, MoKα radiation) were carried out at ∼110(2) K on crystals coated with a thin layer of amorphous oil to minimize crystal deterioration, possible structural disorder, and related thermal motion effects and to optimize the precision of the structural results. These structures were solved by direct and Fourier methods and refined by full-matrix least squares methods (by using standard crystallographic software: SIR97, DIRDIF-96, SHELXTL-2012). All non-hydrogen atoms were refined anisotropically. The hydrogen atoms were located in idealized/calculated positions and were refined by using a riding model. Crystals of SHR-FF peptide was grown by slow evaporation technique, employing slow evaporation of the peptide in phosphate buffer in room temperature. Crystals of diffraction quality were obtained after 2–5 days of sample preparation. CCDC accession no. 1014512.

### Preparation of peptide-DNA complex

SHR-FF^+^ and SHR-FLLF peptide stock was prepared in DMSO (100–200 mg ml^−1^). pEGFP-C2 (1 μg) was mixed with an appropriate amount of peptide to prepare solutions with different N/P ratios and phosphate buffer was added to reach a final volume of 100 μl (pH=7.4). The amount of DMSO in final solution was maintained below 3%. The peptide-DNA solution remained clear during sample preparation as well as after several days of incubation at room temperature. After 0.5–1.5 h of incubation at room temperature, 10 μl of the samples was subjected to electrophoresis through a 1% agarose gel containing two drops of ethidium bromide to visualize DNA using TBE-buffer.

### TEM of peptide-DNA complex

The peptide-DNA complex was incubated for 12 h at room temperature. Aliquots (5 μl) of the samples were placed on 400 mesh copper grids. After 1 min, excess fluids were removed. For negative staining, the grid was stained with 2% uranyl acetate in water and after 2 min, excess fluids were removed from the grid. TEM micrographs were recorded using a JEM-1400 electron microscope (JEOL) operating at 80 kV.

### DNase I digestion assay

SHR-FLLF peptide stock was prepared in DMSO (100–200 mg ml^−1^). pEGFP-C2 (4 μg) was mixed with an appropriate amount of peptide (N/P 100) to reach a final volume of 100 μl in Tris-buffered saline (20 mM Tris HCl, 2 mM MgCl_2_ and 3 mM CaCl_2_, pH=7.4) at 25 °C. The amount of DMSO in final solution was maintained below 3%. DNase I (0.005 U) was added per μg of DNA and incubated at 25 °C. At defined time intervals, 10 μl aliquots of the sample were taken out and mixed immediately with 5 μl EDTA (0.1 M, pH=8) to stop the digestion. The resulting solutions were analysed by electrophoresis on 1% agarose gels. A control experiment with bare DNA was performed under similar condition without the addition of peptide.

## Additional information

**Accession codes**: The X-ray crystallographic coordinates for structures reported in this study have been deposited at the Cambridge Crystallographic Data Centre (CCDC), under deposition numbers CCDC 1014512. These data can be obtained free of charge from The Cambridge Crystallographic Data Centre via www.ccdc.cam.ac.uk/data_request/cif.

**How to cite this article:** Mondal, S. *et al.* Formation of functional super-helical assemblies by constrained single heptad repeat. *Nat. Commun.* 6:8615 doi: 10.1038/ncomms9615 (2015).

## Supplementary Material

Supplementary Information Supplementary Figures 1-10

Supplementary Data 1 Crystal information file
