# Supplementary material for: Formation of functional super-helical assemblies by constrained single heptad repeat
Source: Nat Commun. 2015 Oct 15;6:8615. doi: 10.1038/ncomms9615 (PMC4634320; doi:10.1038/ncomms9615)
Supplement: Supplementary Information — Supplementary Figures 1-10 [file ncomms9615-s1.pdf]

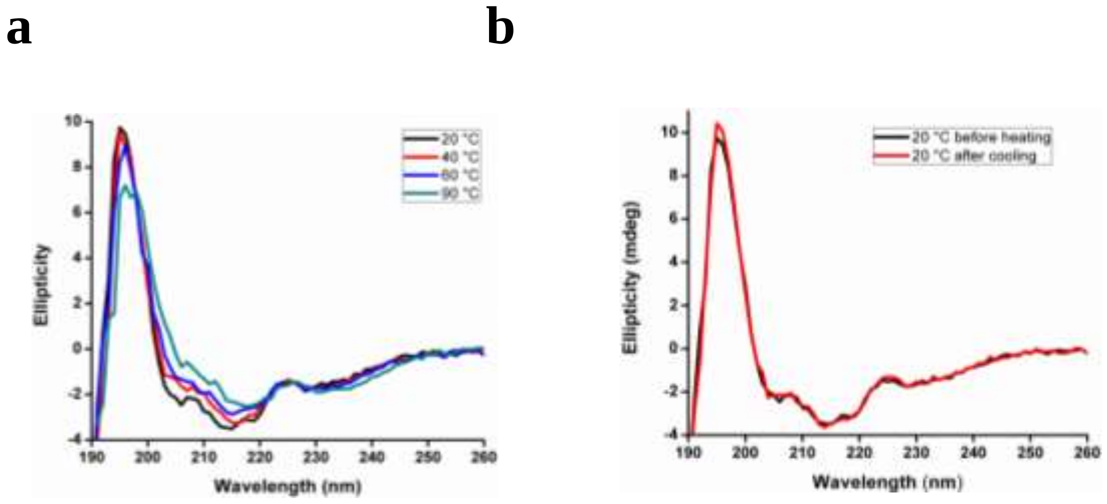

Supplementary Figure 1: Temperature dependent CD spectra of SHR-FF. (a) Spectra were recorded at various temperatures starting from 20 °C and heated to 90 °C. SHR-FF maintains residual helicity even at 90 °C. (b) Spectra of SHR-FF during heating-cooling cycles. A spectrum was recorded at the start of the experiments (20 °C) and the sample was heated to 90 °C followed by cooling to 20 °C. The second spectrum was acquired after equilibrations of the sample at 20 °C for 10 minutes.

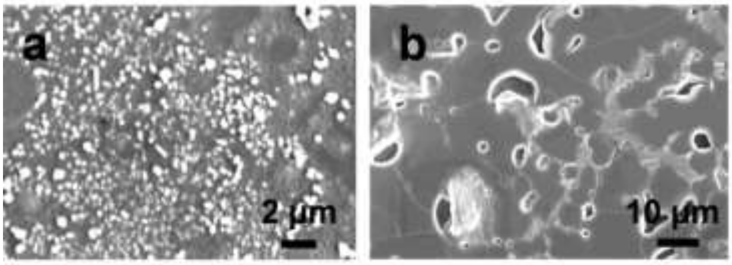

Supplementary Figure 2: **Scanning electron microscopic images of single heptad peptide.** (a) peptide SHR-LL, (b) peptide SHR-FL and (c) peptide SHR-FF.

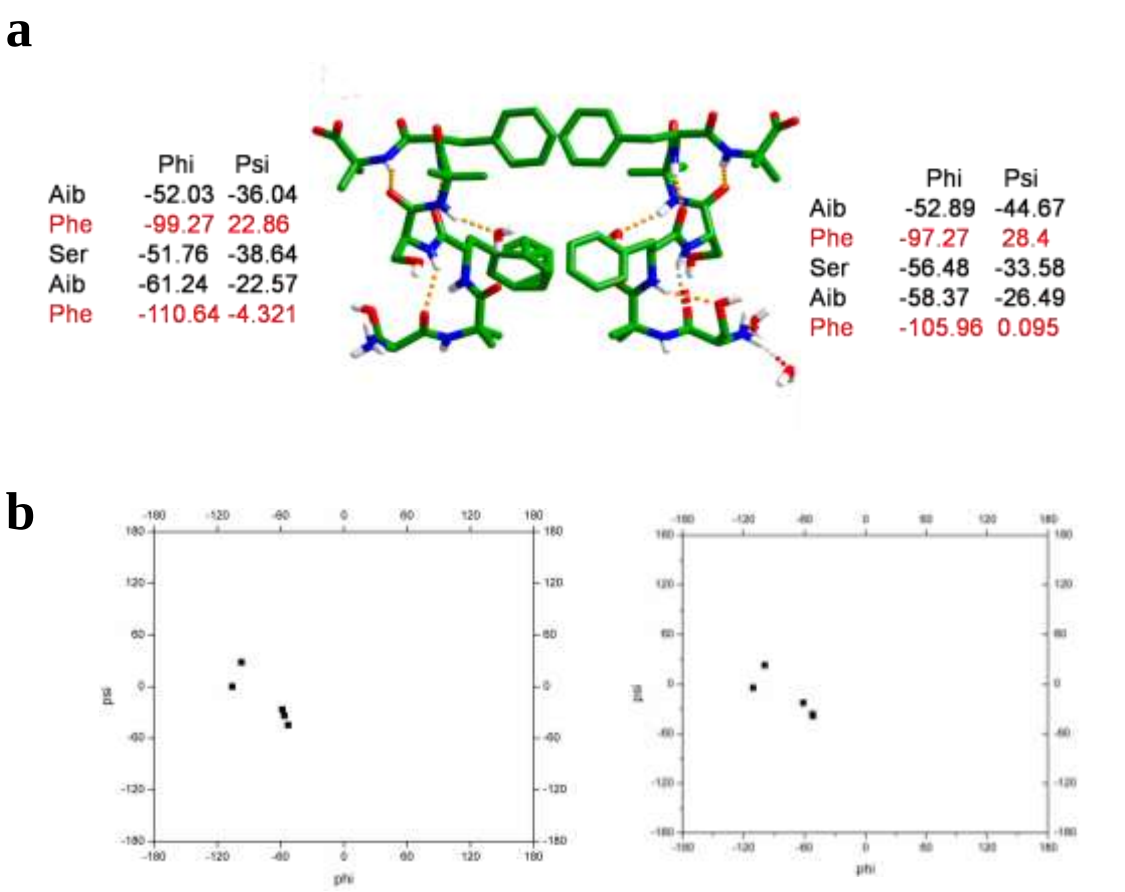

Supplementary Figure 3: Details torsion angles of two asymmetric molecules present in the crystal SHR-FF. (a) Values were reported starting from N-terminus Aib to C-terminus Phe. Left hand column corresponds to the left asymmetric unit and *vice versa*. (b) Ramachandran plot of the dihedral angles.

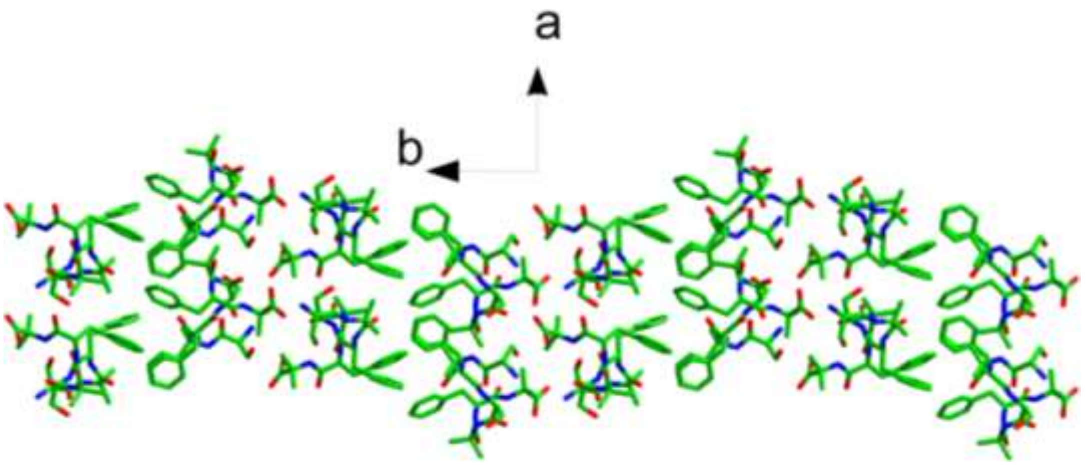

Supplementary Figure 4: View of the two different layers of SHR-FF peptide viewed along crystallographic c-axis**.**

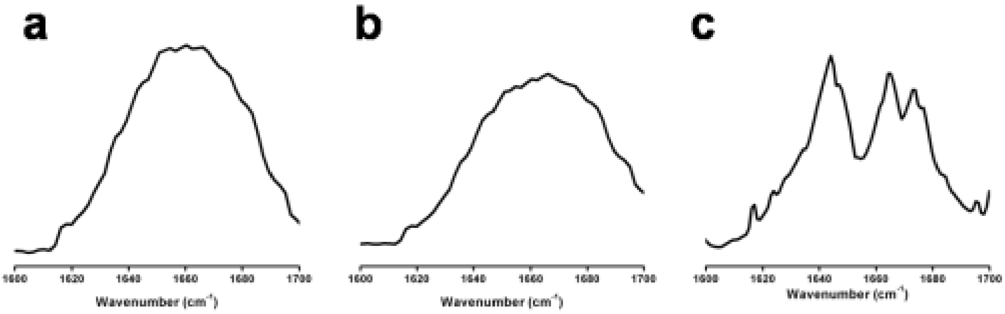

Supplementary Figure 5: FTIR spectra of heptapeptides acquired after deuterium exchange. (a) FTIR spectrum of SHR-LL, (b) FTIR spectrum of SHR-FL, (c) FTIR spectrum of SHR-FF.

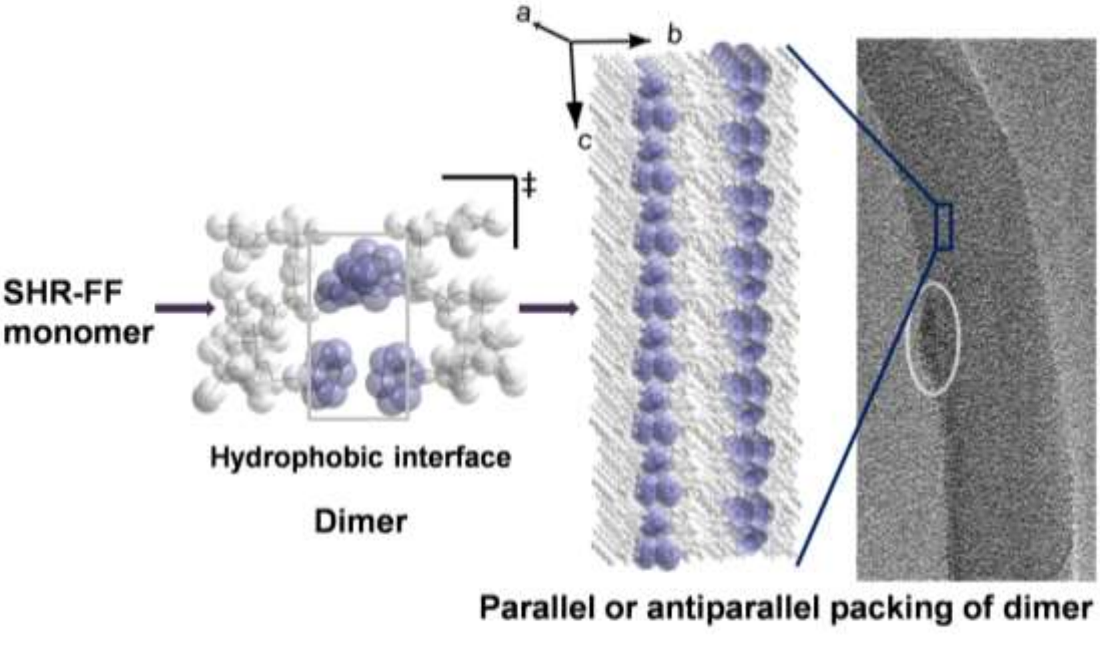

Supplementary Figure 6: Schematic model of SHR-FF peptides self-assembly into fibrillar structures**.**

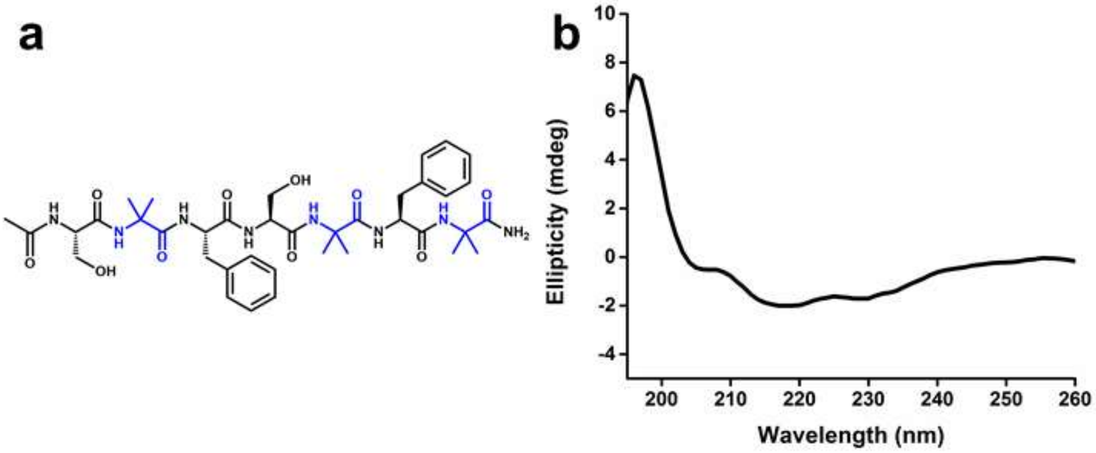

Supplementary Figure 7: (a) Chemical structure and (b) Circular dichroism spectra of Ac-SHR-FF-NH_2

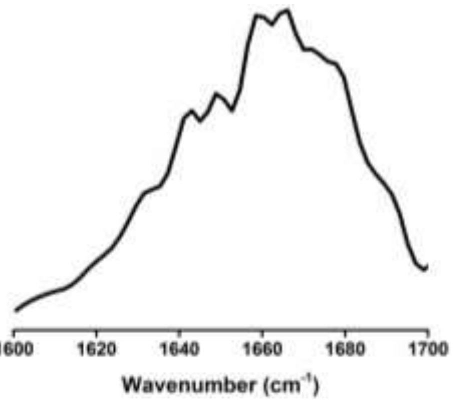

Supplementary Figure 8: FTIR spectra of SHR-FLLF in phosphate buffer (pH = 7.4)**.**

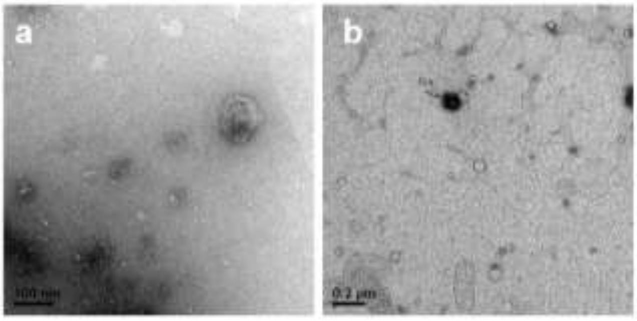

Supplementary Figure 9: TEM images of complexes of plasmid DNA with SHR-FLLF. (a) N/P 20 and (b) N/P 50**.**

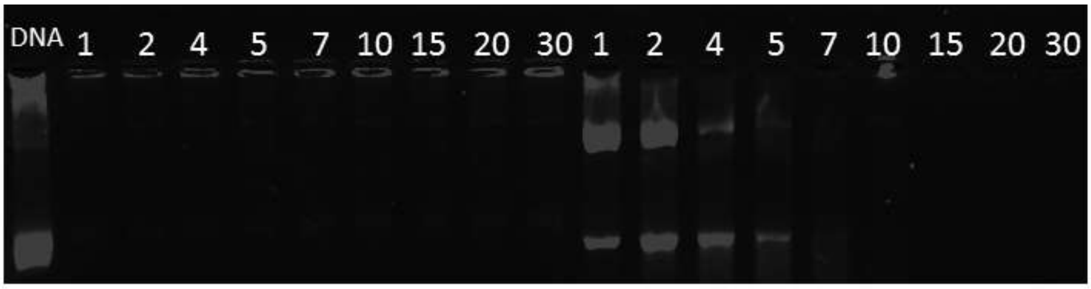

Supplementary Figure 10: DNase I digestion assay. From left to right**;** bare DNA without DNaseI, 1 to 30 represent the incubation time (min) of peptide-DNA (N/P 100) complex with DNase I, 1 to 30 represent the incubation time (min) of bare DNA with DNase I**.**
